# A survey of women diagnosed with breast cancer experiencing oncology treatment–induced hot flushes: identification of specific characteristics as predictors of hot flush occurrence, frequency, and severity

**DOI:** 10.1007/s11764-024-01647-7

**Published:** 2024-07-31

**Authors:** Susan Gallagher, Alice Johnstone, Alysha De Livera, Deborah J. Marsh, Sean Walsh

**Affiliations:** 1 School of Life Sciences, Faculty of Science, University of Technology Sydney, Ultimo, Sydney, New South Wales 2007 Australia; 2 School of Science, RMIT University, Melbourne, VIC Australia; 3 Melbourne School of Population and Global Health, The University of Melbourne, Parkville, VIC Australia; 4 Baker Heart and Diabetes Institute, Melbourne, VIC Australia; 5 Department of Mathematics and Statistics, La Trobe University, Melbourne, VIC Australia; 6 Translational Oncology Group, School of Life Sciences, Faculty of Science, University of Technology Sydney, Ultimo, NSW 2007 Australia; 7 Chinese Medicine Centre, Western Sydney University, Penrith, NSW 2751 Australia; 8 School of Health Sciences, Western Sydney University, Campbelltown, NSW 2560 Australia

**Keywords:** Breast neoplasms/cancer, Hot flushes, Hot flashes, Vasomotor symptoms, Chemotherapy, Hormone therapy, Predictive factors, Survey

## Abstract

**Purpose:**

More women diagnosed with breast cancer (BC) are living with oncology treatment–induced hot flushes (HFs). This Australian-based survey explores why some women experience more severe or ongoing HF and whether specific population characteristics are predictive of HF occurrence, frequency, and/or severity.

**Methods:**

A non-probabilistic anonymous survey distributed online (Register4) and two Australian hospitals collected demographic and clinical information. Eligibility was consenting Australian-based women, 18 years and over, with a primary BC diagnosis. Analysis included linear and logistic regression models.

**Results:**

A total of 324 survey responses were analyzed. Chemotherapy and hormone therapy were each associated with HF occurrence (aOR = 2.92, 95% CI [1.27, 6.70], *p* = 0.01; and aOR = 7.50, 95% CI [3.02, 18.62], *p* < 0.001) and in combination (aOR = 5.98, 95% CI [2.61, 13.69], *p* < 0.001). Increased self-reported anxiety at BC diagnosis was significantly associated with HF frequency and severity scores (aCO = 0.71, 95% CI [0.31, 1.12], *p* = 0.001; and aCO = 0.44, 95% CI [0.33, 0.55], *p* < 0.001). Postmenopausal women had significantly lower HF severity and frequency scores than premenopausal women (aCO = −0.93, 95% CI [−1.62, −0.25], *p* = 0.008; and aCO = −2.62, 95% CI [−5.14, −0.11], *p* = 0.041).

**Conclusions:**

Women with BC receiving chemotherapy and/or hormone therapy and premenopausal or experiencing elevated anxiety and/or stress will likely experience more severe oncology treatment–related HFs.

**Implications for Cancer Survivors:**

HFs continue across the BC treatment trajectory with women >5-year survivorship still reporting life impacts, with premenopausal women at the time of BC diagnosis at higher risk of experiencing severe and more frequent oncology treatment-induced HFs than postmenopausal women. Women at high risk require information on methods to moderate HF potential life impacts and maintain treatment compliance.

**Supplementary Information:**

The online version contains supplementary material available at 10.1007/s11764-024-01647-7.

## Introduction

Breast cancer (BC) is the most common cancer affecting women globally. The occurrence peaks between 55 and 70 years of age, with an estimated 2.3 million women diagnosed in 2020 [1]. In Australia, with earlier detection and improved treatments, the 5-year survival increased from 76.1% in 1988 to 91.5% in 2013 and remains at 91.5% [2], similar to other high-income countries [3].

Between 51 to 82% of women living with a BC diagnosis experience oncology treatment–induced vasomotor symptoms, inclusive of hot flushes (HFs) [4, 5]. BC survivors also experience HFs that are significantly more frequent, severe, distressing, and of greater duration than experienced during menopause [6–8]. Defined as a feeling of intense heat in the face, neck and trunk, HFs last on average 4 min and are accompanied by sweating [9–11]. While menopausal HFs decrease with time, BC treatment–related HFs continue into the post-treatment period [11].

HFs experienced by women with BC negatively impact quality of life (QoL) [12], with sleep disruption, avoidance of social situations and intimate relationships, and decreased performance and ability to work, commonly impacted. They are associated with negative affective states, including anxiety, depression, tension, anger, and confusion [7, 12]. Critically, HFs can interfere with life-saving adherence to prescribed medication [4, 13–15]. Women with a risk of BC relapse have declined hormone therapy in part due to concerns about HFs, while others have discontinued treatment early due to intolerable HFs [12, 16].

Despite the personal and clinical impacts, few studies explore why some women experience more severe or ongoing HF induced from oncology treatment than others. Such information may help manage patients’ expectations and design prospective education and treatment strategies about the effect of HFs to support treatment adherence. The aim of this study was to determine factors associated with BC oncology treatment–induced HFs, and whether specific characteristics are predictive of HF occurrence, frequency, and/or severity.

## Methods

### Study design and inclusion criteria

This is a self-administered, single-measure, anonymous survey using convenience, non-probabilistic sampling conducted in New South Wales (NSW), Australia, with Ethics approval (see below). Survey distribution occurred between January 2020 and September 2021 through NSW-based BC support groups, two large Sydney hospitals (The Kinghorn Cancer Centre, St Vincent’s Hospital and Patricia Ritchie Cancer Care Centre, Mater Hospital), and Register4 (an online community). Eligibility was consenting Australian-based women residing in NSW aged 18 years and over, with a primary BC diagnosis of any stage. There was no time-based restriction since diagnosis. Women with a secondary BC diagnosis were excluded. The survey was administered in English.

There were two survey versions to maximize response: hardcopy and online (via SurveyMonkey) (Appendix A). A copy of the Participant Information Sheet (PIS) was provided electronically via a survey page link before completing the survey. The hardcopy version included the PIS and a reply-paid envelope. The PIS explained the survey purpose that consent was implied through submission of the completed survey, assurance of confidentiality, and time required to complete the survey. No incentive was provided.

### Survey instrument design

A 33-item survey questionnaire was developed after reviewing studies reporting the evaluation of predictive participant characteristics in the occurrence, severity, and/or frequency of HFs (or hot flashes) induced by BC treatment or menopause. Survey questions were generated for the related identified domains: menopausal status, lifestyle factors, cancer treatment and medications, and the occurrence (binary yes/no), frequency (number/day as an aggregate based on recall), and severity of HFs (on a numerical rating scale, where 0 is not severe and 10 is most severe) for women experiencing either natural and/or BC oncology treatment–induced HFs. Generated survey questions explored characteristics in each domain. HF interference on daily activities and QoL for women still experiencing HFs was measured using the validated Hot Flush–Related Daily Interference Scale (HFRDIS) [17]. The Depression Anxiety Stress Scales (DASS-21) assessed respondents’ related negative affective states [18, 19]. The survey was checked for readability, comprehension, flow, and content relevance.

### Data analysis

Data were analyzed using Stata SE 16.0 (StataCorp, College Station, TX, USA). Categorical data are presented as percentages with frequency, and continuous data are presented as mean with standard deviation, while skewed data are presented as median with interquartile range (25th–75th centile). Logistic and linear regression modelling evaluated the relationship between independent variables and the dependent variable of either HF occurrence, frequency, or severity. The linear models were checked for valid assumptions of residual normality, linearity, and homoscedasticity. Multivariable models were examined for multicollinearity using variance inflation factor.

## Results

A total of 328 surveys were returned: 17 paper-based and 311 on-line (Register4, *n* = 260; Mater Hospital, *n* = 40; Kinghorn Cancer Centre, *n* = 11). Register4 provided expression of interest data from 454 women, indicating a 57% response rate. Four surveys not specifying a BC diagnosis were excluded. A final 324 surveys were included for analysis.

### Cohort characteristics

Most participants were over 55 years old (*n* = 235, 73%), with between the ages of 55 and 64 years old (*n* = 112, 35%) being the most frequent age range. Participants more often reported being married or in a *de facto* relationship (*n* = 245, 76%), co-habiting (*n* = 228, 70%), and having children (*n* = 219, 68%). The most frequently indicated nationality at birth was Australian/New Zealander (*n* = 243, 75%), and the most frequently self-reported ethnicity was European (*n* = 173, 54%).

Most women were premenopausal at diagnosis (*n* = 194, 60%) with 81% of these women still experiencing HFs (*n* = 158) compared with 68% (*n* =89) of postmenopausal women. Most women reported a single cancer diagnosis (*n* = 240, 74%), with stage I the most frequent (*n* = 73, 56%). Most women had undergone surgery (*n* = 289, 89%) followed by radiotherapy (*n* = 247, 76%), hormone therapy (*n* = 199, 62%), or chemotherapy (*n* = 194, 60%), with 36% (*n* = 116) reporting combined chemotherapy and hormone therapy. Of the participants prescribed hormone therapy, most specified aromatase inhibitors (AI) (*n* = 95, 73%) followed by tamoxifen (*n* = 36, 27%). Around 23% (*n* = 73) of women completed treatment within the last 2 years, with most (*n* = 187, 60%) completing treatment more than 5 years ago. (Appendix B, Table B.1, summarizes participants’ demographic and oncology-related characteristics.)

### Lifestyle characteristics

Appendix B, Fig. B.1 provides a breakdown of participants’ lifestyle characteristics within each HF occurrence group. Most participants reported low daily dietary intake of vegetables, fruit, and water. Alcohol consumption was mostly “moderate or less” and physical activity was “sufficient.” Approximately a third of the participants had smoked (*n* = 115). A higher percentage of participants who experienced HFs (45%) were a healthy weight (BMI 18.6 to 24.9 kg/m^2^) versus those who did not (36%).

### Instrument-reported outcomes: HFs and stress, anxiety, and depression

The HFRDIS was completed only by women who answered “yes” at Question 32 (survey section 2), indicating they were still experiencing HFs (approx. 60% of respondents still reported HFs). Obtained data were divided into three categories: mild, moderate, and severe. Of the 186 women completing the HFRDIS, 22% (*n* =40) considered HF life interference as moderate or above expectations with sleep the most affected factor (Appendix B, Fig. B2).

All participants irrespective of HF status were asked to complete the self-reported stress/anxiety numerical scale (Question 30) and the DASS-21 (survey section 3; refer to Table 1). Most participants (*n* = 181, 56%) self-reported experiencing above-average levels of stress/anxiety upon commencing cancer treatment (a score of >5). The DASS-21 findings indicated most women were currently within “normal” range for stress (84%), anxiety (78%), and depression (89%) at the time of the survey.

**Table 1:** Outcomes from scales reporting participants’ HF intensity (where relevant) and the cohort’s stress, anxiety, and depression characteristics with and without oncology treatment–induced HFs

Characteristics	All n (%)	With HFs n (%)^	Without HFs n (%)
HFRDIS*	**186 (100%)**	**186 (100%)**	**n/a**
Mild	146 (78.49)	146 (78.49)	**-**
Moderate	29 (15.59)	29 (15.59)	**-**
Severe	11 (5.91)	11 (5.91)	**-**
Self-reported anxiety scale	**324 (100%)**	**247 (76.24%)**	**77 (23.77%)**
≤5	143 (44.14)	135 (41.67)	8 (2.47)
>5	181 (55.86)	112 (34.57)	69 (21.30)
DASS-21 results^^—stress	**314 (100%)**	**238 (75.80%)**	**76 (24.20%)**
Average	264 (84.08)	199 (63.38)	65 (20.70)
>Average	50 (15.92)	39 (12.42)	11 (3.50)
Anxiety	**314 (100%)**	**238 (75.80%)**	**76 (24.20%)**
Average	246 (78.34)	183 (58.28)	63 (20.06)
>Average	68 (21.66)	55 (17.52)	13 (4.14)
Depression	**314 (100%)**	**238 (75.80%)**	**76 (24.20%)**
Average	279 (88.85)	211 (67.20)	68 (21.65)
>Average	35 (11.15)	27 (8.60)	8 (2.55)

^*HFs* oncology treatment–induced hot flushes; *n* number, % calculated out of the total patients in each characteristic group for the scales completed. *HFRDIS Hot Flush–Related Daily Interference Scale. (*n/a*, not applicable) – completed only by women experiencing HFs at the time of the survey. ^^*DASS-21* Depression, Anxiety, and Stress Scale

### Specific characteristics and relationship with HF occurrence

In the univariable regression analysis, which included all women who had ever experienced HFs (Table 2), menopausal status resulted in a statistically significant relationship (*χ*^2^(2) = 6.87, *p* = 0.009). Women who were postmenopausal were less likely to experience HFs compared with premenopausal women (OR = 0.50, 95% CI [0.30, 0.84]).

**Table 2:** Univariable and multivariable models showing the association of significant variables with the occurrence of oncology treatment–induced HFs

Independent variables	Univariable model		Multivariable model^#^	
	OR (95% CI)	*p-*value	OR (95% CI)	*p-*value
Age				
≤34	Reference		Reference	
35–44	1.47 (0.35, 6.16)	0.593	1.06 (0.23, 4.73)	0.935
45–54	1.57 (0.38, 6.39)	0.526	1.14 (0.26, 5.07)	0.854
55–64	1.20 (0.29, 4.91)	0.796	1.15 (0.22, 5.97)	0.861
65–74	0.34 (0.08, 1.46)	0.151	0.34 (0.06, 1.91)	0.224
Education level				
Academic/university degree	Reference		Reference	
Apprenticeship/diploma	1.33 (0.70, 2.56)	0.386	1.53 (0.74, 3.16)	0.245
High school graduate or less	0.53 (0.29, 1.00)	0.049*	0.86 (0.41, 1.80)	0.698
Menopausal status prior to cancer				
Premenopausal	Reference		Reference	
Postmenopausal	0.50 (0.30, 0.84)	0.009*	0.74 (0.32, 1.72)	0.489
Oncology treatment				
Neither chemo- nor hormone	Reference		Reference	
Chemotherapy	4.05 (1.86, 8.81)	0.000*	2.92 (1.27, 6.70)	0.011*
Hormone therapy	7.65 (3.32, 17.61)	0.000*	7.50 (3.02, 18.62)	0.000*
Chemo- and hormone therapy	8.19 (3.75, 17.88)	0.000*	5.98 (2.61, 13.69)	0.000*

*OR* odds ratio, *CI* confidence interval, Reference: baseline value

The dependent variable in this analysis is occurrence of oncology treatment–induced HFs coded

0 = no and 1 = yes. ^#^Model adjusted for the variables shown in the table. *Significant *p*-value (<0.05)

A statistically significant association of HFs with chemotherapy (OR = 4.05, 95% CI [1.86, 8.81]), hormone therapy (OR = 7.65, 95% CI [3.32, 17.61]), and a combination of chemotherapy and hormone therapy (OR = 8.19, 95% CI [3.75, 17.88]) was also found.

Multiple logistic regression was undertaken on the data of *n* = 316 respondents with predictors selected based on a literature review (adjusted age, education level, menopausal status prior to cancer and oncology treatment). The model showed statistical significance for chemotherapy (aOR = 2.92, 95% CI [1.27, 6.70], *p* = 0.011), hormone therapy (aOR = 7.50, 95% CI [3.20, 18.62], *p* < 0.001), and combined chemotherapy and hormone therapy (aOR = 5.98, 95% CI [2.61, 13.69], *p* < 0.001) which remained significant predictors of HF occurrence.

### Specific characteristics and relationship with HF frequency and severity

Univariable regression results between specific characteristics and HF are shown in Table 3 (HF frequency) and Table 4 (HF severity). Chemotherapy was associated with HF frequency (CO = 2.26, 95% CI [0.27, 4.29], *p* = 0.030) and hormone therapy was negatively associated (CO = −2.69, 95% CI [−4.77, −0.6], *p* = 0.012). Post-menopausal status was associated with less frequent and less severe HFs than premenopausal status (respectively, CO = −3.27, 95% CI [−5.27, −1.26], *p* = 0.002 and CO = −1.22, 95% CI [−1.83, −0.63], *p* < 0.001). Higher self-reported anxiety upon commencing cancer treatment was associated with an increase in HF frequency and severity (respectively, CO = 0.87, 95% Cl [0.50, 1.24], *p* < 0.001; and CO = 0.46, 95% CI [0.36, 0.56], *p* < 0.001), as was completing an apprenticeship or awarded a diploma (respectively, CO = 2.39, 95% CI [0.17, 4.62], *p* = 0.035; and CO = 0.72, 95% CI [0.03, 1.40], *p* = 0.041); however, only marginally compared to having a university level degree.

**Table 3:** Univariable and multivariable models showing the associations between variables and oncology treatment–induced HF frequency

Independent variables	HF frequency (*n* = 241): univariable model		HF frequency (*n* = 217): multivariable model^#^	
	CO (95% CI)	*p*-value	CO (95% CI)	*p*-value
Education level				
Academic/university degree	Reference		Reference	
Apprenticeship/diploma	2.39 (0.17, 4.62)	0.035	2.04 (−0.29, 4.38)	0.086
High school graduate/less	2.31 (−0.43, 5.06)	0.098	2.44 (−0.55, 5.44)	0.109
Menopausal status prior to cancer				
Premenopausal	Reference		Reference	
Postmenopausal	−3.27 (−5.27, −1.26)	0.002*	−2.62 (−5.14, −0.11)	0.041*
Oncology treatment				
Neither chemo- nor hormone	Reference		Reference	
Chemotherapy	2.26 (0.27, 4.29)	0.030*	1.07 (−1.20, 3.35)	0.352
Hormone therapy	−2.69 (−4.77, −0.60)	0.012*	−2.32 (−4.55, −0.08)	0.043*
Radiotherapy	0.87 (−1.50, 3.23)	0.472	1.9 (−0.53, 0.34)	0.125
Surgery	0.37 (−3.01, 3.75)	0.829	−1.15 (−4.89, 2.59)	0.546
Self-reported anxiety scale	0.87 (0.50, 1.24)	0.000*	0.71 (0.31, 1.12)	0.001*
DASS-21 results (*n* = 147)				
Stress	0.19 (−0.11, 0.48)	0.206		
Anxiety	0.46 (0.05, 0.87)	0.028*		
Depression	0.18 (−0.16, 0.52)	0.296		

*HF* hot flush, *CO* coefficient, *CI* confidence interval, *DASS-21* Depression, Anxiety, and Stress Scale, Reference: baseline value. ^#^Model adjusted for the variables shown in the table. *Significant *p*-value (<0.05)

**Table 4:** Univariable and multivariable models showing the associations between variables and oncology treatment–induced HF severity

Independent variables	HF severity (*n* = 241): univariable model		HF severity (*n* = 217): multivariable model^#^	
	CO (95% CI)	*p*-value	CO (95% CI)	*p*-value
Education level				
Academic/university degree	Reference		Reference	
Apprenticeship/diploma	0.72 ( 0.03, 1.40)	0.041*	0.38 (−0.25, 1.02)	0.238
High school graduate/less	0.56 (−0.27, 1.04)	0.186	0.31 (−0.52, 1.13)	0.463
Menopausal status prior to cancer				
Premenopausal	Reference		Reference	
Postmenopausal	−1.22 (−1.83, −0.63)	0.000*	−0.93 (−1.62, −0.25)	0.008*
Oncology treatment				
Chemotherapy	0.38 (−0.25, 1.01)	0.237	0.09 (−0.53, 0.70)	0.784
Hormone therapy	−0.09 (−0.75, 0.56)	0.779	0.26 (−0.35, 0.87)	0.398
Radiotherapy	0.33 (−0.40, 1.07)	0.368	0.66 (0.00, 1.33)	0.05
Surgery	0.05 (−1.00, 1.11)	0.919	−0.89 (−1.91, 0.13)	0.086
Self-reported anxiety scale	0.46 (0.36, 0.56)	0.000*	0.44 (0.33, 0.55)	0.000*
DASS-21 results (*n* = 147)				
Stress	0.12 (0.04, 0.21)	0.005*		
Anxiety	0.23 (0.12, 0.35)	0.000*		
Depression	0.07 (−0.02, 0.17)	0.135		

*HF* hot flush, *CO* coefficient, *CI* confidence interval, *DASS-21* Depression, Anxiety, and Stress Scale, Reference: baseline value. ^#^Model adjusted for the variables shown in the table. *Significant *p*-value (<0.05)

Premenopausal women experienced more severe and frequent HFs when commencing oncology treatment (Appendix B: Fig. B.3 and B.4). As self-reported anxiety increased, so did the HF severity score, which showed a positive moderate linear relationship (Pearson’s correlation = 0.51, *p* < 0.001). An increase in self-reported anxiety escalated with HFs’ daily frequency; however, it was a weak linear relationship with many outliers (Pearson’s correlation = 0.30, *p* < 0.001) (Appendix B: Fig. B.5 and B.6).

A sub-analysis comparing the HFRDIS data of 147 women (that is, those still experiencing HFs at completion of the survey) with their complete DASS-21 responses was undertaken. Anxiety had a significant association with HF frequency (Table 3) and severity (Table 4) (respectively, CO = 0.46, 95% CI [0.05, 0.87], *p* = 0.028; and CO = 0.23, 95% CI [0.12, 0.35], *p* < 0.001). Stress had a significant association with HF severity (CO = 0.12, 95% CI [0.04, 0.21], *p* = 0.005) (Table 4).

In the multiple linear regression, the confounders of age, menopausal status, living arrangement, educational level, BMI, alcohol consumption, oncology treatment, and anxiety were included in the analysis based on the literature reviewed. The model for severity was statistically significant (*R*^2^ = 0.34, *F*(18,198) = 5.69, *p* < 0.001), as was the model for frequency (*R*^2^ = 0.18, *F*(18,198) = 2.46, *p* = 0.0013). Hormone therapy and postmenopausal status were only marginally significant with HF frequency respectively (aCO = −2.32, 95% CI [−4.55, −0.08], *p* = 0.043; and aCO = −2.62, 95% CI [−5.14, −0.11], *p* = 0.041). However, menopausal status remained significant with HF severity (aCO = −0.9; 95% CI [−1.62, −0.25], *p* = 0.008). Self-reported anxiety upon cancer diagnosis was also significant with HF frequency (aCO = −0.71, 95% CI [0.31, 1.12], *p* = 0.001) and HF severity (aCO = 0.44, 95% CI [0.33, 0.55], *p* < 0.001).

## Discussion

This is the first Australian-based survey completed by women with a BC diagnosis reporting their experiences of oncology treatment*–*induced HFs. Having chemotherapy and/or hormone therapy was statistically significantly associated with the occurrence of treatment-induced HFs. Increased anxiety was associated with increased severity and frequency of HFs, with premenopausal women being most at risk, especially for HF severity.

Women who received chemotherapy had 2.9-fold higher odds of HFs while those taking hormone therapy had 7-fold higher odds, compared to women receiving neither chemotherapy nor hormone therapy (Table 2), with premenopausal women at more risk. For chemotherapy, this is a similar outcome to the results reported by Reeves et al. [20] who noted an approximate 2-fold higher odds of HFs (OR 1.80, 95% CI [1.21–2.68]). However, Reeves et al. [20] only reported approximately 2-fold higher odds of HFs in those who received hormone therapy (OR 2.73, 95% CI [2.08*–*3.58]) compared with 7-fold higher increase in our study. The higher rate may reflect cohort menopausal status differences with Reeves et al. exclusively recruiting postmenopausal women for whom hormonal disruption may be less marked compared with premenopausal women (who comprised 60% of participants in our study). Lastly, only 62% of women indicated they received hormonal therapy which is low considering approximately 75% of all BC are estrogen receptor positive [21]. The proportion of estrogenic receptor positive BC participants was unknown in the present study.

A longitudinal study by Savard et al. [22] of 126 women reported chemotherapy associated with greater HF severity and frequency compared to women who had received radiotherapy or a healthy control group. Severity saw a 13.2-percentile difference in the combined scores recorded in a HF diary between the chemotherapy (94.8) and radiotherapy (81.6) groups 3 months post-treatment. Our results indicated chemotherapy was marginally associated with a higher HF frequency only in the univariable analysis, and not associated with HF severity. Variance in data collection time points may account for differences with our study, with the effect of treatment on HFs diminishing over time. Additionally, we found no significant association between radiotherapy and HFs. Both AI and tamoxifen were associated with the occurrence of HFs.

Menopausal status was significantly related to HF occurrence when considered as a univariate model but was not significant when controlling for chemotherapy, hormone therapy, and confounders. Other studies report that younger and premenopausal women generally receive more intense chemotherapy/hormone therapy [4, 20, 23–25], which may confound the results.

In a cohort study involving 5023 Chinese women with BC, Dorjgochoo et al. [23] reported the highest occurrence of HFs was in the 46 to 55 years age range, the oldest group of premenopausal women. This is similar to the current study, even though the two studies had differences in respondent ethnicity, the odds of treatment-induced HFs were highest between the ages of 45 and 54. Menopausal status was significantly associated with HF severity (*p* = 0.008) and associated with HF frequency (*p* = 0.041). Few studies have examined HF frequency and severity, and further investigation is required.

According to the World Health Organization, 60% of factors related to individual health and QoL are correlated to lifestyle choices [26]. Caffeinated drinks and alcohol are established precipitators of HFs and avoiding these may help to decrease them [27]. Evidence indicates that BMI above the normal range (>25 kg/m^2^) increases the likelihood of HFs in both oncology [4, 22] and menopause populations [28, 29]. While BMI differences of 10% (Fig. B.1) were noted between women experiencing and those not experiencing HF in this study, this was not significant and not explored further.

A cancer diagnosis is a catalyst for anxiety and it is reasonable to consider the formation of a compounding reciprocating relationship with HFs. Llaneza et al. [30] proposed that an increase in sympathetic activity associated with episodes of stress and anxiety exacerbates oncology treatment–induced HFs. Menopausal HF research reported women with moderate or high anxiety were respectively nearly three and five times more likely to report HFs compared to women with mild levels of anxiety [28]. A study of 56 French Canadian women by Guimond et al. [31] found greater anxiety predicted more severe self-reported HFs. A statistically significant relationship was found in this study between self-reported anxiety upon cancer diagnosis and both severity (aCO = 0.44, 95% CI [0.33, 0.55], *p* < 0.001) and frequency (aCO = 0.71, 95% CI [0.31, 1.12], *p* = 0.001) of treatment-induced HFs; however, Pearson’s correlation coefficient indicates only a moderate relationship with severity (*r* = 0.5), and a weak relationship with frequency (*r* = 0.3). While anxiety influenced the impact of HFs, there was no association found with HF occurrence in the current study (refer to Table 1). It is possible that associations were underestimated or undetected as the self-reported anxiety scale required participants to rate their anxiety retrospectively, which may lead to recall bias.

To examine associations with stress, anxiety, and depression using the DASS-21, the sample was reduced to women who were experiencing treatment-induced HFs at the time of participating in the survey (*n* = 146). Anxiety was associated with HF severity and frequency, and stress was associated with HF severity in the univariate analysis. No statistically significant association was found with depression. The nature of the association is not clear—whether HF experience increases stress and anxiety or whether stress and anxiety trigger HFs.

There were study limitations. Firstly, 187 participants (59%) received a BC diagnosis > 5 years ago, with 78% (*n* = 146) continuing to experience HFs. This means the reported HF experiences may be weighted towards current happenings rather than those experienced at the time of their original diagnosis. Additionally, there is recall bias relating to past experiences of HFs at the time of cancer diagnosis. For this reason, the HFRDIS was only completed by women still experiencing HFs. For questions with open responses, there were instances of incomplete information, miss-spelling, and response avoidance due to possible uncertainty. Self-reported ethnicity under-represented women from culturally and linguistically diverse communities and further reach-out to overcome coverage bias is required. Finally, the study occurred during the COVID-19 pandemic and moved online to ensure population reach. Moving forward, further study is recommended using a repeat-measure study design to track HF occurrence, frequency, and severity relative to women’s experience across the BC treatment and survivorship trajectory.

## Conclusion

Women between 45 and 55 years of age diagnosed with BC who received chemotherapy and/or hormone therapy alone and in combination or were anxious or stressed experienced greater issues with oncology treatment–induced HFs. Women sharing these characteristics in the future should receive focused support to mediate the life impacts of HFs. We also found HFs continue for women across the trajectory of survivorship, with 78% of women >5 years of survivorship still reporting life impacts. Different from previous studies, we identified premenopausal women at diagnosis at higher risk of severe and more frequent HF compared to postmenopausal women (aCO = −0.93; 95% CI [−1.62, −0.25], *p* = 0.008) and (aCO= −2.62, 95% CI [−5.14, −0.11], *p* = 0.041) respectively.

Knowing the predictive impact of oncology treatment–induced HFs has implications for the targeted use of finite health resources and improves the success of reducing adverse impacts while helping treatment compliance by supporting those women most at risk. There are additional ethical and consensual considerations to inform women diagnosed with BC in advance of treatment about the considerable impact of HFs. Finally, oncology treatment–induced HFs effect several domains of women’s lives, and further investigation into therapies to moderate or alleviate the considerable impacts is required.

## Supplementary information

ESM 1 Appendix A: Survey instrument (DOCX 1802 kb)

ESM 2 Appendix B: Participant demographics (Table B.1), Lifestyle characteristics (Fig B.1), Hot Flush Related Daily Interference Scale (HFRDIS) (Fig. B.2), HF frequency and severity figures (Fig. B.3-B.6) (DOCX 230 kb)

## Data Availability

The datasets analyzed during the current study are available from the corresponding author (Deborah J. Marsh) on reasonable request.

## Abbreviations

AI: Aromatase inhibitor
aOR: Adjusted odds ratio
aCO: Adjusted coefficient
BC: Breast cancer
BMI: Body mass index
CI: Confidence interval
CO: Coefficient
DASS-21: Depression Anxiety Stress Scales
HFRDIS: Hot Flush–Related Daily Interference Scale
HFs: Hot flushes
HREC: Human Research Ethics Committee
OR: Odds ratio
PIS: Participant Information Sheet
QoL: Quality of life
UTS: University of Technology Sydney
VMS: Vasomotor symptoms
