# Supplementary material for: A survey of women diagnosed with breast cancer experiencing oncology treatment–induced hot flushes: identification of specific characteristics as predictors of hot flush occurrence, frequency, and severity
Source: J Cancer Surviv. 2024 Jul 31;20(1):209–17. doi: 10.1007/s11764-024-01647-7 (PMC12906599; doi:10.1007/s11764-024-01647-7)
Supplement: Supplementary file 1 — Appendix A: Survey instrument (DOCX 1802 kb) [file 11764_2024_1647_MOESM1_ESM.docx]

Appendix A: Survey Tool
