# Supplementary material for: A survey of women diagnosed with breast cancer experiencing oncology treatment–induced hot flushes: identification of specific characteristics as predictors of hot flush occurrence, frequency, and severity
Source: J Cancer Surviv. 2024 Jul 31;20(1):209–17. doi: 10.1007/s11764-024-01647-7 (PMC12906599; doi:10.1007/s11764-024-01647-7)
Supplement: Supplementary file 2 — Appendix B: Participant demographics (Table B.1), Lifestyle characteristics (Fig B.1), Hot Flush Related Daily Interference Scale (HFRDIS) (Fig. B.2), HF frequency and severity figures (Fig. B.3-B.6) (DOCX 230 kb) [file 11764_2024_1647_MOESM2_ESM.docx]

Appendix B:

**Table B.1.** Cohort characteristics of survey participants with and without oncology treatment-induced HFs. Percentages (%) shown in relation to the number of participants (n) in the sub-group breakdown.

| **Characteristics** | **All   n (%)** | **With ^HFs**  **n (%)** | **Without ^HFs   n (%)** | |
| --- | --- | --- | --- | --- |
| **Age (years):**  ≤44  45–54  55–64  65-74  ≥75  **Marital status**  Never Married  Married/De facto Divorced/separated  Widowed  **Living arrangements**  Alone  Dependents  Co-habiting  **Children**  Yes  No  **Dependents**  Adult  <18 years old  Mixed  None  **Self-reported ethnicity**  Australian/New Zealander  European  Mixed Ethnicity  Other (Asian, Middle Eastern, South American, South African, Polynesian)  **Nationality at birth**  Australian/New Zealander  European  Other (diverse mix)  **Education level**  High school graduate or less  Apprenticeship/diploma  Academic/University degree  **Career**  Professional  Non-professional  Unknown  **Employment status**  Full-time and Self employed  Part-time and Casual worker  Unemployed  Homemaker  Retired  Medically Retired | **323 (100%)**  23 (7.12)  65 (20.12)  112 (34.67)  92 (28.48)  31 (9.60)  **323 (100%)**  19 (5.88)  245 (75.85)  45 (13.93)  14 (4.33)  **324 (100%)**  50 (15.43)  46 (14.20)  228 (70.37)  **323 (100%)**  219 (67.80)  104 (32.20)  **323 (100%)**  157 (48.61)  43 (13.31)  19 (5.88)  104 (32.20)  **322 (100%)**  66 (20.50)  173 (53.73)  74 (22.98)  9 (2.79)  **322 (100%)**  243 (75.47)  61 (18.94)  18 (5.59)  **324 (100%)**  65 (20.06)  90 (27.78)  169 (52.16)  **324 (100%)**  246 (75.93)  61 (18.83)  17 (5.25)  **320 (100%)**  77 (24.06)  82 (25.63)  7 (2.19)  13 (4.06)  129 (40.31)  12 (3.75) | **246 (76.16%)**  18 (5.57)  59 (18.27)  92 (28.48)  62 (19.20)  15 (4.64)  **246 (76.16%)**  16 (4.95)  190 (58.82)  32 (9.91)  8 (2.48)  **247 (76.23%)**  34 (10.49)  39 (12.04)  174 (53.70)  **246 (76.16%)**  169 (52.32)  77 (23.84)  **246 (76.16%)**  118 (36.53)  34 (10.53)  17 (5.26)  77 (23.84)  **245 (76.09%)**  46 (14.29)  135 (41.93)  56 (17.39)  8 (2.48)  **246 (76.40%)**  184 (57.14)  48 (14.91)  14 (4.35)  **247 (76.23%)**  42 (12.96)  74 (22.84)  131 (40.43)  **247 (76.23%)**  187 (57.72)  48 (14.81)  12 (3.70)  **243 (75.93%)**  67 (20.94)  65 (20.31)  6 (1.88)  13 (4.06)  82 (25.62)  10 (3.12) | **77 (23.84%)**  5 (1.55)  6 (1.86)  20 (6.19)  30 (9.29)  16 (4.95)  **77 (23.84%)**  3 (0.93)  55 (17.03)  13 (4.02)  6 (1.86)  **77 (23.77%)**  16 (4.94)  7 (2.16)  54 (16.67)  **77 (23.84%)**  50 (15.48)  27 (8.36)  **77 (23.84%)**  39 (12.07)  9 (2.79)  2 (0.62)  27 (8.36)  **77 (23.91%)**  20 (6.21)  38 (11.80)  18 (5.59)  1 (0.31)  **76 (23.60%)**  59 (18.32)  13 (4.04)  4 (1.24)  **77 (23.77%)**  23 (7.10)  16 (4.94)  38 (11.73)  **77 (23.76%)**  59 (18.21)  13 (4.01)  5 (1.54)  **77 (24.06%)**  10 (3.12)  17 (5.31)  1 (0.31)  0 -  47 (14.69)  2 (0.62) |  |
| **Natural menopause HFs**  **(absence of intervention)**  Hot flushes  No hot flushes  **Menopausal status at diagnosis**  Premenopausal  Postmenopausal | **165 (100%)**  9 (5.45)  156 (94.55)  **324 (100%)**  194 (59.88)  130 (40.12) | **121 (73.34%)**  6 (3.64)  115 (69.70)  **247 (76.24%)**  158 (48.77)  89 (27.47) | **44 (26.67%)**  3 (1.82)  41 (24.85)    **77 (23.76%)**  36 (11.11)  41 (12.65) |  |
| **Total No. of cancer diagnoses**  1  2  3  **Breast Cancer Stage (most advanced specified)**  I  II-III  IV | **324 (100%)**  240 (74.07)  68 (20.99)  16 (4.94)  **131 (100%)**  73 (55.73)  48 (36.64)  10 (7.63) | **247 (76.24%)**  180 (55.56)  54 (16.67)  13 (4.01)  **106 (80.91%)**  54 (41.22)  42 (32.06)  10 (7.63) | **77 (23.76%)**  60 (18.52)  14 (4.32)  3 (0.93)  **25 (19.08%)**  19 (14.50)  6 (4.58)  0 - | |
| **Total Breast Cancer Interventions***  Surgery  Radiotherapy  Chemotherapy  Hormone Therapy  Chemo & Hormone Therapy  Neither Chemo nor Hormone | 289 (89.47)  247 (76.47)  194 (60.06)  199 (61.61)  116 (35.91)  46 (14.24) | 223 (69.04)  191 (59.13)  157 (48.61)  169 (52.32)  99 (30.65)  19 (5.88) | 66 (20.43)  56 (17.34)  37 (11.46)  30 (9.29)  17 (5.26)  27 (8.36) | |
| **Hormone Therapy Type**  Tamoxifen  Aromatase Inhibitor | **131 (100%)**  36 (27.48)  95 (72.52) | **116 (88.55%)**  32 (24.43)  84 (64.12) | **15 (11.45%)**  4 (3.05)  11 (8.40) | |
| **Interval between diagnosis and completion of survey**  <6 months (within treatment)  <2 years  <5 years  >5 years | **317 (100%)**  18 (5.67)  55 (17.35)  57 (17.98)  187 (58.99) | **243 (76.66%)**  13 (4.10)  42 (13.25)  42 (13.25)  146 (46.06) | **74 (23.34%)**  5 (1.60)  13 (4.10)  15 (4.73)  41 (12.93) | |

^HFs: oncology treatment-induced hot flushes; n=number, % calculated out of the total patients in each characteristic group. *Showing multiple interventions within the same respondent – counts across row for each intervention by HF status.

**Fig. B.1** A breakdown of HF occurrence within participants’ lifestyle characteristics.

HFs: hot flushes; Body Mass Index: BMI; Percentages calculated within each HF occurrence group

Hot Flush Related Daily Interference Scale (HFRDIS)

Fig B.2 The boxplots show the range (min and max score as tail) and distribution (box indicating 25^th^, 50^th^ and 75^th^ percentile) of participant scores measuring the degree in which HFs interfere with nine daily activities and overall QoL. Outliers for each category are indicated by a filled circle (>1.5 IQR). HF: Hot Flush

HF frequent and severity univariate analysis

Simple linear regression was used to analyse the relationship between the independent variables of menopausal status, level of anxiety at diagnosis, oncology treatment (chemotherapy and hormone therapy) and hot flush frequency and severity.

|  |  |
| --- | --- |
| **Fig. B.3** Comparison of the average predicted severity score. Participants were asked to score the severity of their HFs on a scale between 0-10, (with 10 being the most severe). The data were used to calculate a predicted average score for oncology treatment-induced HFs in pre-and postmenopausal women with breast cancer (n=241). | **Fig. B.4** Comparison of the average predicted daily number of oncology treatment-induced HFs. Participants were asked to estimate the number of HFs they experienced daily. The data were used to calculate a predicted average daily frequency of HFs in pre-and postmenopausal women with breast cancer (n=241). |
| 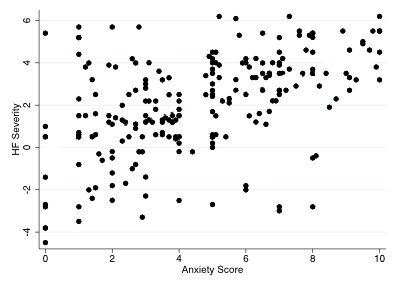 | 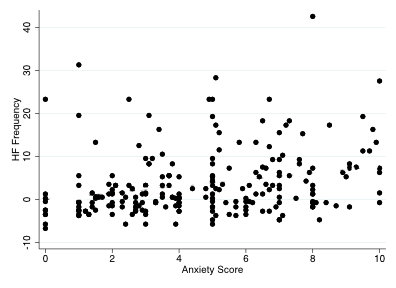 |
| **Fig. B.5** Plots on relationship between anxiety (x-axis) and HF severity (y-axis) using participants self-reported estimates of anxiety at the time of diagnosis (0-10, with 10 being the most severe) and HF severity 0-10 (with 10 being the most severe). The residuals had a standard variance across the range of values for anxiety with no notable trend. | **Fig. B.6** Plots on the relationship between anxiety (x-axis) and HF frequency (y-axis) using participants self-report estimates of anxiety at the time of diagnosis (0-10, with 10 being the most severe) and the number of daily HFs (frequency estimate). The residuals had a standard variance across the range of values for anxiety with no notable trend. |
